# The First Study of *Borrelia burgdorferi* Sensu Lato Persistence in Small Mammals Captured in the *Ixodes persulcatus* Distribution Area in Western Siberia

**DOI:** 10.3390/pathogens14121200

**Published:** 2025-11-24

**Authors:** Vera Rar, Valeriy Yakimenko, Yana Igolkina, Yuliya Sabitova, Valeria Fedorets, Alfrid Karimov, Gavril Rubtsov, Tamara Epikhina, Nina Tikunova

**Affiliations:** 1 Institute of Chemical Biology and Fundamental Medicine SB RAS, 630090 Novosibirsk, Russia; igolkina@inbox.ru (Y.I.); yvsabitova@mail.ru (Y.S.); v.fedorets@alumni.nsu.ru (V.F.); tikunova@1bio.ru (N.T.); 2 Omsk Research Institute of Natural Foci Infections, 644080 Omsk, Russia; vyakimenko78@yandex.ru (V.Y.);

**Keywords:** *Borrelia bavariensis*, *Borrelia afzelii*, “*Candidatus* Borrelia sibirica”, *Ixodes* spp., voles, mixed infection, persistence, transmission, reservoir competence, phylogenetic analysis

## Abstract

*Borrelia burgdorferi* sensu lato (s.l.) persistence in reservoir hosts is essential for the maintenance of the spirochaetes in the enzootic cycle. In this study, we investigated the persistence of Siberian *B. burgdorferi* s.l. strains in naturally infected voles and their transmission to *Ixodes* ticks. A long-term study conducted in 2013–2024 demonstrated the presence of *Borrelia afzelii*, *Borrelia bavariensis*, and, rarely, “*Candidatus* Borrelia sibirica” DNA in blood samples of small mammals. Among these, *B. bavariensis* exhibited the highest genetic diversity. All identified *Borrelia* species persisted in naturally infected *Clethrionomys* spp. voles throughout their lifespan (up to 50 weeks), providing the first evidence of long-term persistence of *B. bavariensis* and “*Candidatus* B. sibirica” in these hosts. Notably, the persistence of two *Borrelia* genospecies or several genovariants of a single genospecies within the same vole was common. Xenodiagnosis with laboratory-reared *Ixodes* spp. confirmed efficient transmission of all identified *Borrelia* genospecies to *Ixodes persulcatus* after 35–42 weeks of *B. burgdorferi* s.l. persistence. Moreover, *B. bavariensis* was transmitted to *Ixodes pavlovskyi* and *I. persulcatus*/*I. pavlovskyi* interspecies hybrids after at least 23 weeks of pathogen persistence. These findings demonstrate the reservoir competence of *Clethrionomys* spp. for *B. afzelii*, *B. bavariensis,* and “*Candidatus* B. sibirica”.

## 1. Introduction

Spirochetes of the *Borrelia burgdorferi* sensu lato (s.l.) species complex are widely distributed throughout the Northern Hemisphere. Among the 20 accepted and three proposed genospecies of this species complex, at least eight species are known to cause Lyme borreliosis (LB) in humans [1,2,3,4]. Of these, the most epidemiologically significant are *Borrelia burgdorferi* sensu stricto (s.s.), inhabiting mainly North America, as well as *Borrelia afzelii*, *Borrelia bavariensis*, and *Borrelia garinii*, which are widely distributed in Eurasia; these species are the most extensively studied [1,5,6].

The maintenance of *B. burgdorferi* s.l. in natural foci depends critically on its persistence in reservoir hosts, as these spirochetes are transmitted horizontally from infected vertebrate hosts to ticks but not transovarially. Small rodents and birds are the most common reservoir hosts. Birds are the main reservoirs for *B. garinii*, while small mammals are the main hosts for *B. burgdorferi* s.s., *B. afzelii*, and *B. bavariensis* [1,7,8,9,10,11]. The efficiency of this enzootic cycle relies on the ability of larval ticks to acquire the spirochetes from infected hosts and subsequently transmit them, as nymphs, to susceptible hosts. In contrast to immature stages, adult ticks play an auxiliary role as vectors because they feed primarily on large animals that are incompetent reservoir hosts [12,13]. In addition to systemic transmissions, *B. burgdorferi* s.l. can be effectively transmitted directly between ticks during simultaneous feeding on hosts in the absence of systemic infection (co-feeding transmission) [14].

The persistence of *B. burgdorferi* s.l. in small mammals and their transmission to ticks was studied in detail for US strains of *B. burgdorferi* s.s. and European strains of *B. afzelii* [15,16,17,18,19]. It has been shown that different strains within the same genospecies significantly vary in the duration of persistence in wild and laboratory mice and efficiency of systemic and co-feeding transmission [12,20]. Thus, some strains can persist in small mammals during their lifetime (up to 40 months) with high transmission efficiency, whereas other strains are rapidly cleared [21,22]. For some strains, the efficiency of systemic transmission decreased from the acute to chronic phase of infection [17]. Notably, co-feeding transmission was shown to be effective for rapidly cleared strains, while systemic transmission is more characteristic of strains with prolonged persistence [23].

Local populations of *B. burgdorferi* s.s. and *B. afzelii* usually contain multiple genetically diverse strains [15,24,25,26]. Up to ten genovariants of *B. burgdorferi* s.s. were found in a single *I. scapularis* tick [26] and up to six strains of *B. afzelii* were detected in a single individual vole [16], indicating that coinfection of several strains in the rodent hosts in nature is common. Some laboratory experiments demonstrated that competition between strains can reduce the host-to-tick transmission of both strains [27]. However, in other experiments, the asymmetry in competition can lead to the extinction of a less competitive strain [28].

In the Asian part of Russia, *B. afzelii*, *B. bavariensis*, and *B. garinii* are the most prevalent *B. burgdorferi* s.l. genospecies [6,29]. *Borrelia afzelii* and *B. bavariensis* are associated with *Ixodes persulcatus* ticks, whereas *B. garinii* is more frequently detected in the *Ixodes pavlovskyi* distribution area [29,30,31]. In addition, *B. valasianna* was detected in single *I. persulcatus* ticks [29,32]. Notably, a new proposed *Borrelia* genospecies with unknown pathogenicity, “*Candidatus* Borrelia sibirica”, was found in small mammals and ticks feeding on them in several *I. persulcatus*/*I. trianguliceps*/*I. apronophorus* sympatric areas of Western Siberia [33]. Since “*Candidatus* B. sibirica” has not been detected outside the *I. apronophorus* distribution area to date, it is assumed that *I. apronophorus* is the most likely vector for this candidate genospecies.

In the *I. persulcatus* distribution area in the Asian part of Russia, the prevalence of *B. burgdorferi* s.l. in small mammals has been examined in only a limited number of studies [33,34,35]. It was shown that the overall prevalence of *B. burgdorferi* s.l. in small mammals can exceed 50% in some years and that *B. burgdorferi* s.l. population was presented mainly by *B. afzelii* and *B. bavariensis*, with “*Candidatus* B. sibirica” detected only rarely.

European and Asian populations of *B. bavariensis* are genetically distinct [6]. To date, the reservoir competence of small mammals has been established for European, but not Asian, *B. bavariensis* genotypes [10]. Moreover, the long-term persistence of *B. bavariensis* in small mammals has not been studied for either European or Asian genotypes. Data on the persistence of “*Candidatus* B. sibirica” in small mammals is also lacking.

In this study, we investigated the role of small mammals as reservoir hosts for *Borrelia* genospecies circulating in the *I. persulcatus* distribution area of Western Siberia. The main objectives of the research were to determine the prevalence and genetic diversity of *B. burgdorferi* s.l. in small mammals, examine *B. burgdorferi* s.l. persistence in naturally infected voles, and evaluate the reservoir competence of *Clethrionomys* spp. voles for *Borrelia* genospecies circulating in the studied area using xenodiagnosis.

## 2. Materials and Methods

### 2.1. Sampling

All experiments with animals were conducted in compliance with the Animal Welfare Act at the Omsk Research Institute of Natural Foci Infections, according to the guidelines for experiments with laboratory animals (Supplement to the Order of the Russian Ministry of Health, no. 755, of 12 August 1977). This study was approved by the Bioethical Committee of the Omsk Research Institute of Natural Foci Infections (Protocol No.1, 20 March 2013; Protocol No. 4, 17 February 2016; Protocol No.1, 15 March 2024).

**Figure 1:**
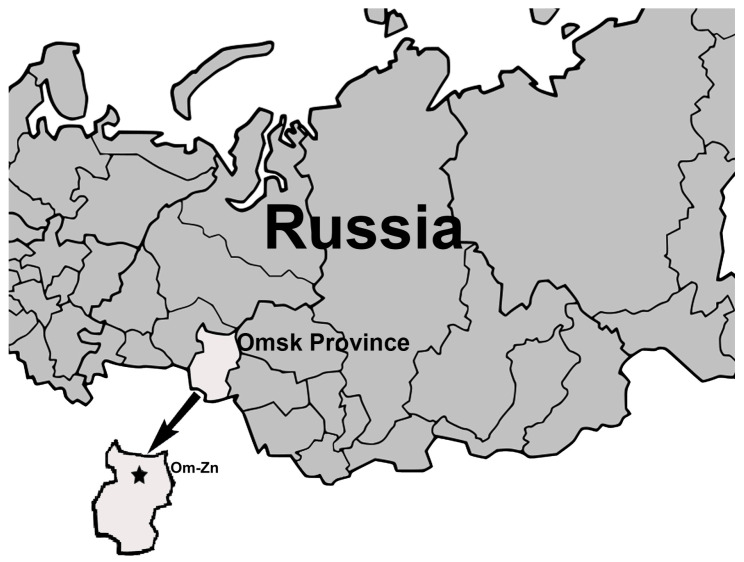
The map shows the location of a sampling site.

The study area, covering approximately 20 km^2^ (57°23′ N, 73°40′ E), was located in the Znamenskiy district of the Omsk province, Western Siberia, Russia, within the southern taiga subzone of the forest landscape zone (Figure 1). The landscape is characterized by continuous tracts of deciduous, mixed, and coniferous forests, predominantly pine. The terrain includes elevated areas and depressions featuring swampy birch and coniferous forests, as well as small transitional swamps.

Small mammals were captured during eleven sampling periods (2–4 weeks per period) from June to October in 2013–2018 and 2024 using live traps with standard bait—brown bread, saturated in unrefined sunflower oil, as previously described [36]. Between 50 and 150 traps were deployed in lines at 5-m intervals and inspected twice daily. A total of 150–300 traps were used per day, which corresponds to 1200–2500 trap-days per sampling period. Species identification was based on morphological characteristics, including body size, coat and tail coloration, and tail length [37,38]. A single blood sample was collected from each vole. Following initial sampling, animals were either labeled by ear tags or transported to the laboratory for further study. Blood samples (100 μL/animal) were taken from the lateral saphenous veins, collected into sterile tubes with 15 μL of 0.5 M EDTA, mixed with 200 μL lysis buffer (4 M guanidine thiocyanate, 0.1 M Tris−HCl pH 6.4, 0.045 M EDTA pH 8.0, and 1.3% Triton X-100), and stored at 4–8 °C until DNA extraction.

For subsequent examination of *B. burgdorferi* s.l. long-term persistence and their transmission to ticks, a number of sexually mature *Clethrionomys* spp. voles were transported to the laboratory. To minimize further pathogen exposure, the animals were manually cleared of all ectoparasites with fine tweezers prior to the study.

### 2.2. The Study of B. burgdorferi s.l. Persistence

In the laboratory, captured voles were kept individually in plastic cages with standard bedding under a natural light cycle throughout their lives. They received water and a balanced diet containing grains, vegetables, and legumes, supplemented with vegetable oil, ad libitum. Blood samples were taken from voles as described in Section 2.1 with intervals from 1 to 5 weeks and examined for the presence of *B. burgdorferi* s.l. DNA.

### 2.3. Laboratory Colonies of Ticks

The study of *B. burgdorferi* s.l. transmission was conducted by xenodiagnosis using laboratory colonies of ticks. The colony 5 of *I. pavlovskyi* and the colony 7/8–14 of *I. persulcatus* were obtained from ticks collected in Novosibirsk province in May 2013 and Omsk province in May 2014. Tick colonies were maintained for three and four generations, respectively, at 24–26 °C and ~100% humidity using standard methods [39]. To obtain interspecies hybrids, *I. persulcatus* females and *I. pavlovskyi* males from the second tick generations were crossed. To prevent conspecific mating, ticks were isolated individually upon reaching the engorged nymph stage. To verify the species identity of the tick colonies, a subset of larval offspring were genetically characterized for the mitochondrial cytochrome c oxidase subunit 1 (*cox*1) gene and nuclear multi-copy internal transcribed spacer (ITS2), as described previously (Table 1) [29,40]. The resulting larvae were stored at 4–8 °C until transmission experiments.

### 2.4. The Study of B. burgdorferi s.l. Transmission

Larvae from laboratory colonies of ticks were fed on *Clethrionomys* spp. voles until repletion. Up to 30 larvae were fed simultaneously on one animal. The engorged larvae were placed in containers and maintained at a temperature of 24–26 °C and ~100% humidity until molting. After molting, most nymphs were maintained under the same conditions for two weeks, then frozen and stored at −80 °C until DNA extraction.

In addition, several *I. pavlovskyi* nymphs were maintained at 24–26 °C and ~100% humidity for 110–140 days, after which they were fed on two-week-old white mice until repletion and maintained at 24–26 °C for three months until molting or the onset of morphogenetic diapauses. The ticks were then frozen and stored at −80 °C until DNA extraction.

### 2.5. DNA Extraction

To prevent cross-contamination, DNA extraction, PCR assay, and electrophoresis were conducted in separate rooms. Molted ticks were washed in bi-distilled water, 70% ethanol, and bi-distilled water and homogenized with a MagNA Lyser system (Roche Diagnostics, Basel, Switzerland). Total DNA was extracted from blood samples and homogenized ticks using the Proba NK kit (DNA-Technology, Moscow, Russia) according to the manufacturer’s protocol.

### 2.6. Detection and Genetic Characterization of B. burgdorferi s.l.

Detection of *B. burgdorferi* s.l. was carried out using nested PCR with primer sets flanking the 5S-23S rRNA intergenic spacer (IGS) of *B. burgdorferi* s.l. (Table 1). “*Candidatus* B. sibirica” was detected by nested reactions with species-specific primers by the IGS region (Table 1). The fragments of *clp*A and *p83/100* genes were amplified using nested PCR with primers specified in Table 1. All PCR reactions were performed in 20 μL of the reaction mixture containing 1× PCR buffer, 200 μM of each dNTP, 2U of Taq DNA polymerase (Biolabmix, Novosibirsk, Russia), 0.5 μM of primers, and 2 μL of DNA for primary reactions or 2 μL of the primary PCR products for nested reactions. PCR protocol includes initial denaturation at 94 °C for 3 min followed by 35 cycles of denaturation at 94 °C for 0.5 min, annealing at temperatures specified in Table 1 for 0.5 min, and elongation at 72 °C for 1 min; final elongation was conducted at 72 °C for 5 min. Sterile bi-distilled water was used as a negative control. DNA of *B. afzelii* (str. Tom1303) and *B. garinii* (str. Tom3005) was used as a positive control. To distinguish *B. afzelii* from *B. bavariensis* and identify cases of mixed infections, the lengths of obtained PCR fragments of the *p83/100* gene were compared; the lengths of *B. afzelii* and *B. bavariensis* fragments were 336 bp and 426 bp, respectively [29]. Genetic characterization for a number of positive specimens from molted ticks and blood samples was conducted by sequencing the *clp*A and *p83/100* gene fragments in both directions.

### 2.7. Phylogenetic Analysis

The obtained PCR fragments were purified in 0.6% SeaKem^®^ GTG-agarose (Lonza, Haifa, Israel). Sanger sequencing was conducted using BigDye Terminator V. 3.1 Cycling Sequencing Kit (Applied Biosystems, Carlsbad, CA, USA). Sanger reaction products were analyzed using an ABI 3500 Genetic Analyzer (Applied Biosystems, Carlsbad, CA, USA). The determined *clp*A and *p83/100* gene sequences were compared with those available on the NCBI website using the BLASTN (https://blast.ncbi.nlm.nih.gov/Blast.cgi), accessed on 23 September 2025. In addition, the determined *clp*A gene sequences were analyzed using the PubMLST website (https://pubmlst.org/organisms/borrelia-spp), accessed on 23 September 2025. Phylogenetic trees were constructed using the Maximum likelihood (ML) method based on the Tamura-Nei model in MEGA 7.0 with 1000 bootstrap replicates [42].

### 2.8. Statistical Analysis

Statistical analysis was performed to compare the prevalence of *B. burgdorferi* s.l. genospecies in different hosts. Minimum infection rate (MIR) was calculated as the ratio of the number of positive pools to the total number of examined species. The 95% confidence intervals (CI) were computed using an Excel spreadsheet (http://www.pedro.org.au/english/downloads/confidence-interval-calculator/, accessed on 23 September 2025). Differences in the prevalence of infectious agents in different hosts were computed using the Pearson χ^2^ goodness-of-fit test (http://www.socscistatistics.com/tests/chisquare/, accessed on 23 September 2025). *p* < 0.05 was regarded as significant.

### 2.9. GenBank Accession Numbers

Nucleotide sequences determined in the study are available in the GenBank database under accession numbers: PX455120-PX455198.

## 3. Results

### 3.1. B. burgdorferi s.l. Prevalence in Small Mammals

A total of 737 small mammals, including 356 *Clethrionomys rutilus*, 210 *Clethrionomys glareolus*, 93 *Clethrionomys rufocanus*, 70 *Microtus agrestis*, 2 *Microtus oeconomus*, 4 *Apodemus agrarius*, and 2 *Sorex araneus*, were captured at a single site in Omsk province during 11 sampling periods between 2013 and 2018, and again in 2024 (Figure 1, Table 2). Blood samples were collected immediately after trapping and screened for the presence of *B. burgdorferi* s.l. DNA.

*Borrelia burgdorferi* s.l. DNA was found in 83/737 (11.3%) of blood samples; in different periods, the pathogen prevalence varied from 4.2% to 40.6% (Table 2). Analysis by host species revealed infection in 51 of 356 (14.3%) *Cl. rutilus*, 19 of 210 (9.0%) *Cl. glareolus*, 10 of 93 (10.8%) *Cl. rufocanus*, and 1 of 70 (1.4%) *Mi. agrestis*. Among the less common species, *B. burgdorferi* s.l. was identified in one *Mi. oeconomus* and one *S. araneus*, but was not detected in *Ap. agrarius* (Table S1). The prevalence of *B. burgdorferi* s.l. in each of the *Clethrionomys* species was significantly higher than in *Mi. agrestis* (*p* < 0.05); however, there were no significant differences in *Borrelia* prevalence among the different *Clethrionomys* species.

The genospecies of the identified spirochetes were successfully determined for 77 *B. burgdorferi* s.l. samples using species-specific PCR and/or sequencing. DNA of *B. afzelii*, *B. bavariensis*, and “*Candidatus* B. sibirica” were found in 33, 35, and 1 sample, respectively. Additionally, eight samples contained mixed DNA of both *B. afzelii* and *B. bavariensis* (Table 2).

### 3.2. Persistence of B. burgdorferi s.l. in Naturally Infected Voles

To study the long-term persistence of *B. burgdorferi* s.l. in naturally infected voles, the mature *Clethrionomys* spp. voles captured in July and September 2015 were periodically examined for the infection with *B. burgdorferi* s.l. The study group consisted of 47 voles (36 *Cl. rutilus*, 6 *Cl. rufocanus,* and 5 *Cl. glareolus*) that survived for at least seven weeks post-capture. The group included a similar proportion of males (55.3%) and females (44.7%) (Table 3). Blood samples were collected at 1- to 5-week intervals throughout the animals’ lives, with a maximum monitoring period of 50 weeks.

A total of approximately 740 blood samples were collected from the 47 voles, representing 8 to 25 samples per individual. All samples were screened for *B. burgdorferi* s.l. DNA. Infection was detected in 22 of the 47 voles (46.8%), comprising 17 *Cl. rutilus*, 3 *Cl. glareolus*, and 2 *Cl. rufocanus* (Table 3). The prevalence of infection was similar between males (12/26, 46.2%) and females (10/21, 47.6%).

**Figure 2:**
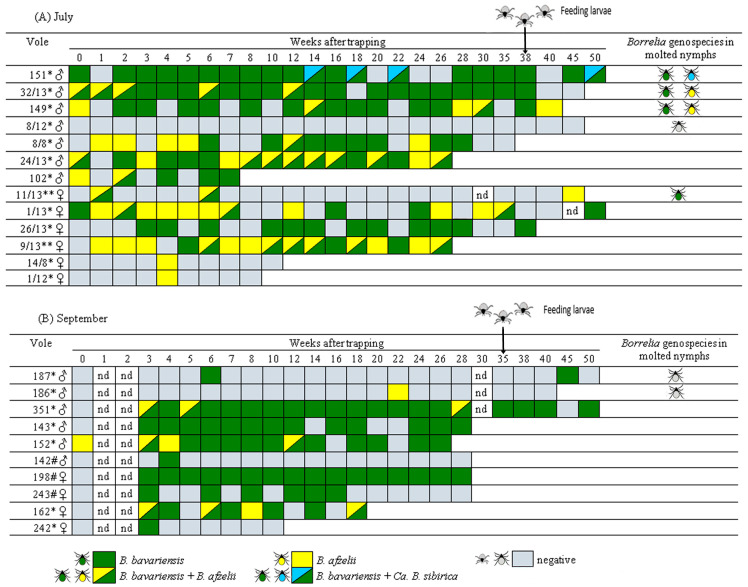
Persistence of *B. burgdorferi* s.l. in naturally infected voles and their transmission to *Ixodes* ticks. PCR-positive vole blood samples and molted nymphs are marked by color: *B. bavariensis* by green, *B. afzelii* by yellow, “*Candidatus* B. sibirica” by blue. PCR-negative blood samples, molted nymphs, and larvae are marked in grey. *—*Clethrionomys rutilus*; **—*Clethrionomys rufocanus*; #—*Clethrionomys glareolus*; nd—not determined.

Among the PCR-positive voles, the proportion of positive samples per individual varied widely, from 5.0% to 94.4%. This distribution was polarized: a majority of infected voles (14 of 22; 63.6%) exhibited a high proportion of positive samples (>50%), while a substantial subset (7 of 22; 31.8%) showed a very low proportion (<15%) (Table 3). In individual voles, positive samples appeared randomly distributed over time, with no clear trend toward pathogen clearance (Figure 2). Notably, *B. burgdorferi* s.l. DNA was still detectable 50 weeks after initial capture in three of the five surviving voles.

Genospecies of *B. burgdorferi* s.l were determined for all 224 PCR-positive samples. *Borrelia bavariensis* was the most prevalent, identified in 146 samples, followed by *B. afzelii* detected in 34 samples. Mixed infections were also common, with 36 samples co-infected with *B. afzelii* and *B. bavariensis*, and four with “*Candidatus* B. sibirica” and *B. bavariensis* (Table 3). When including mixed infections, the overall prevalence of *B. bavariensis* was significantly higher than that of *B. afzelii* in blood samples from voles captured in both July and September (*p* < 0. 001). Furthermore, the prevalence of *B. bavariensis* was significantly higher in blood samples from voles captured in September (70/82, 85.4%) than in those from July (80/142, 56.3%; *χ*^2^ = 19.8, *df* = 1, *p* < 0.001). Conversely, infections with *B. afzelii* alone, as well as mixed *B. afzelii*/*B. bavariensis* infections, were significantly more frequent in the July group (Table 3). Similarly, the proportion of voles infected exclusively with *B. bavariensis* was higher in September, although this difference was not statistically significant due to the small sample size in the groups compared (Table 3, Figure 2).

### 3.3. Genetic Diversity of B. burgdorferi s.l. in Voles

**Figure 3:**
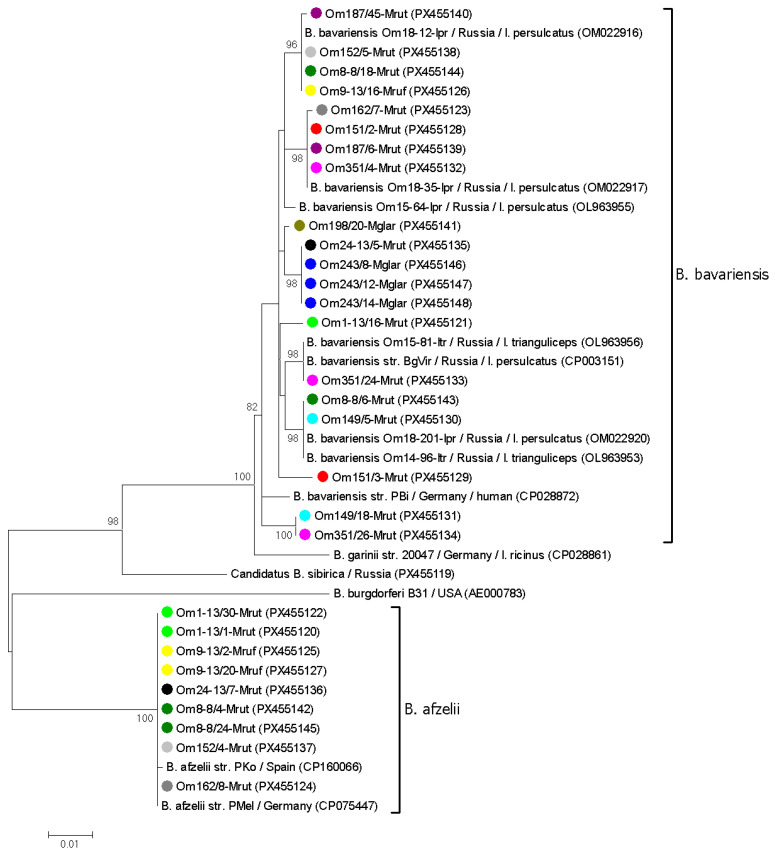
The phylogenetic tree was constructed by the ML method based on nucleotide sequences of a 775 bp fragment of the *clp*A gene of *Borrelia burgdorferi* s.l. identified in blood samples of voles. Sample names include the vole identifier, time post-capture (in weeks), and host species. Different blood samples from the same individual vole are color-coded. The scale bar indicates an evolutionary distance of 0.01 nucleotide per position in the sequence. Significant bootstrapping values (>70%) are shown on the nodes. *B. burgdorferi* B31 was used as an outgroup.

Randomly selected *B. afzelii* and *B. bavariensis* samples from 13 voles were genotyped by sequencing *clp*A gene fragments. Only 2 from 20 *B. afzelii* sequences contained a single polymorphic site; the remaining sequences matched the *clp*A allele 36 in the PubMLST database. In contrast, the *clp*A fragments of *B. bavariensis* were more variable. In total, 18 of the 38 *B. bavariensis* sequences contained from 1 to 6 polymorphic sites, indicating the simultaneous persistence of multiple genovariants. Among the remaining sequences, eleven distinct *B. bavariensis* genovariants were identified. Eight of these genovariants corresponded to known sequences in the PubMLST database (*clp*A alleles 60, 69, 72, 144, 197, and 207) and GenBank database (e.g., OM022917, PX097757). In addition, sequences from samples Om162/7-Mrut, Om187/45-Mrut, and Om198/20-Mglar were novel, differing by 1–3 substitutions from the closest sequences from *I. persulcatus* in Omsk province (OM022917, OM022916, and OL963955) (Figure 3).

**Figure 4:**
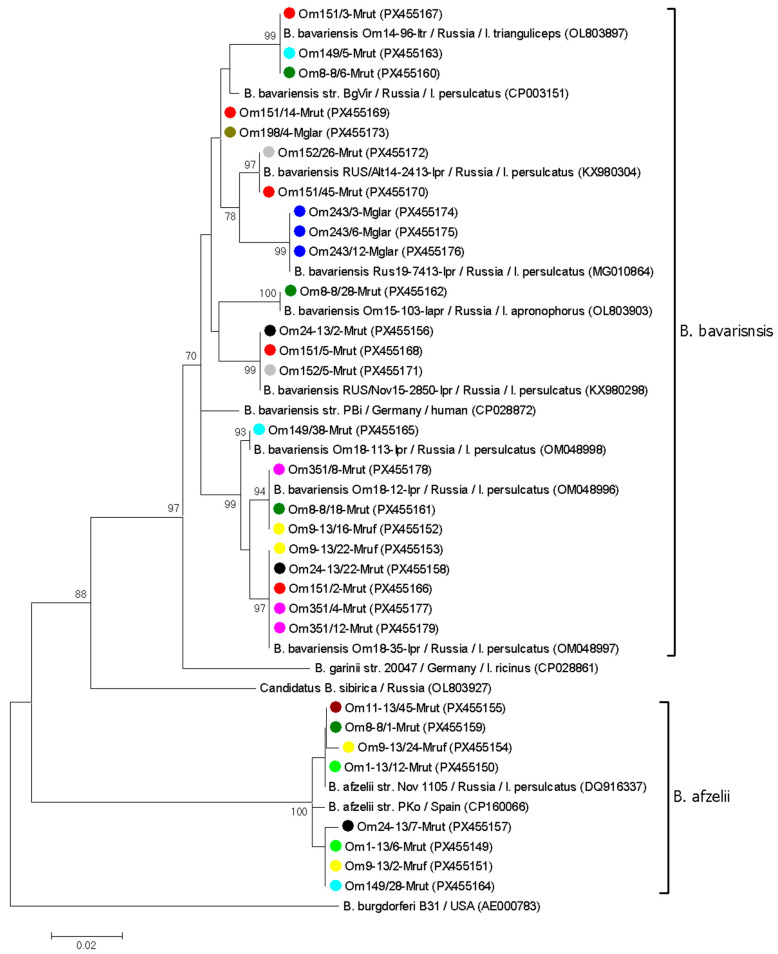
The phylogenetic tree constructed by the ML method based on nucleotide sequences of 276–366 bp fragments of the p83/100 gene of *Borrelia burgdorferi* s.l. identified in blood samples of voles. Sample names include the vole identifier, time post-capture (in weeks), and host species. Different blood samples from the same individual vole are color-coded. The scale bar indicates an evolutionary distance of 0.02 nucleotide per position in the sequence. Significant bootstrapping values (>70%) are shown on the nodes. *B. burgdorferi* B31 was used as an outgroup.

Similarly, a number of *B. afzelii* and *B. bavariensis* samples from twelve voles were genetically characterized based on *p83/100* gene fragments. Four sequence variants, differing by 1–4 nucleotide substitutions, were identified among eight *B. afzelii* sequences. Two variants corresponded to common *B. afzelii* genovariants previously determined in *I. persulcatus* from Western Siberia (OM022930, DQ916337). The sequences from two other samples (Om9-13/24-Mruf, Om24-13/7-Mrut) differed from the known *B. afzelii* sequences by a single nucleotide substitution (Figure 4).

Consistent with the *clp*A genotyping results, *B. bavariensis* exhibited greater diversity in the *p83/100* gene. In total, 22 of 45 *B. bavariensis p83/100* gene sequences contained 1–14 polymorphic sites per sequence. The remaining 23 *B. bavariensis* sequences corresponded to nine genovariants, eight of which matched sequences previously reported in *Ixodes* spp. from Western Siberia (OM048997, OL803903, KX980298, OM048996, PX117462, KX980304, OM048998, and OL803897). Sequences from two samples (Om151/14-Mrut and Om198/4-Mglar) differed from their closest sequence (CP003151) by one substitution each (Figure 4).

Only sequences without mixed infections were included in the phylogenetic analysis of the *clp*A and *p83/100* gene fragments. In the resulting trees, the obtained *B. afzelii* and *B. bavariensis* sequences, along with corresponding reference sequences, formed distinct, well-supported clades (Figure 3 and Figure 4). In most cases, *B. burgdorferi* s.l. sequences from the same vole host represented different genospecies or genovariants within a genospecies. For example, two different *B. afzelii* variants and two *B. bavariensis* genovariants based on the *p83/100* gene were identified in samples from vole 9/13 (Figure 4). An exception was vole 243, for which all tested PCR-positive blood samples contained the same *B. bavariensis* genovariant for both the *clp*A and *p83/100* genes (Figure 3 and Figure 4).

### 3.4. Transmission of B. burgdorferi s.l. by Ixodes spp.

The transmission of *B. burgdorferi* s.l. by *Ixodes* spp. ticks was assessed using xenodiagnosis with larvae from laboratory colonies of *I. persulcatus*, *I. pavlovskyi*, and their first-generation (F1) hybrids. A subset of larvae from each colony was genotyped based on the ITS2 and *cox1* gene to confirm species identification.

To investigate the transmission of *B. burgdorferi* s.l. by *I. persulcatus*, laboratory-reared larvae were fed on seven voles at 35–42 weeks post-capture. All these voles were used in a prior persistence study (Section 3.2); they exhibited varying proportions of PCR-positive samples and were infected with different combinations of *Borrelia* genospecies: (i) *B. bavariensis* and “*Candidatus* B. sibirica” (vole #151), (ii) *B. afzelii* and *B. bavariensis* (voles #149 and #32/13), (iii) *B. afzelii* (vole #186), (iv) *B. bavariensis* (vole #187). Vole #8–12, which had no PCR-positive blood samples, served as a negative control (Figure 2, Table 4).

Engorged larvae that successfully molted to nymphs (116 of 240 fed larvae) were tested for the presence of *B. burgdorferi* s.l. DNA. The resulting nymphs were examined individually (5 ticks) or in 21 pools of 4–7 ticks each. DNA of *B. burgdorferi* s.l. was detected in nymphs that had fed, as larvae, on four voles. The minimum infection rate (MIR) for *B. burgdorferi* s.l. was 18.8–22.2% for ticks that fed on voles #151, #149, and #32/13 (which had 74–88% PCR-positive blood samples) and 10.0% for ticks that fed on vole #11/13 (which had 13% positive blood samples). No transmission was detected for ticks that fed on voles with 5–9% positive blood samples or on the uninfected control vole (Figure 2, Table 4).

The *Borrelia* genospecies detected in molted nymphs matched those found in the blood samples of the voles on which they had fed as larvae (Figure 2, Table 4). Among molted nymphs, *B. bavariensis* was the most frequently detected, with an MIR of 10.0–22.2% across ticks from different voles. This prevalence was higher than that of *B. afzelii* (MIR: 7.1–11.1%) and *“Candidatus* B. sibirica” (MIR: 7.1%). However, the difference in genospecies prevalence among ticks that fed on the same individual vole was not statistically significant (*p* > 0.1).

The transmission of *B. burgdorferi* s.l. to *I. pavlovskyi* and *I. persulcatus*/*I. pavlovskyi* hybrids (F1) was investigated using two *Cl. rutilus* voles (#6 and #24), captured in October 2017. Both voles tested positive for *B. bavariensis* DNA in all three analyzed blood samples; however, a detailed study of *B. bavariensis* persistence in these voles was not conducted. At 23 weeks post-capture, separate groups of 30 *I. pavlovskyi* and 30 hybrid larvae were fed on individual voles. Of these, 15 *I. pavlovskyi* and 14 hybrid larvae successfully molted to nymphs. A subset of these nymphs was individually screened for *B. burgdorferi* s.l. DNA, revealing *B. bavariensis* in three of six (50.0%) *I. pavlovskyi* and five of seven (71.4%) hybrid nymphs (Table 4).

For subsequent transmission experiments, a group of the molted *I. pavlovskyi* nymphs was used. At 110–140 days post-larval feeding, these nymphs were fed until repletion on two-week-old naïve laboratory mice and then maintained at room temperature for three months. During this period, four engorged nymphs molted successfully into adults (two males and two females), while three nymphs failed to molt and entered diapause. *Borrelia bavariensis* DNA was detected in one resulting female (Om24-Ipavl-F) and one engorged nymph (Om24-Ipavl-N-eng); the remaining ticks were PCR-negative.

**Figure 5:**
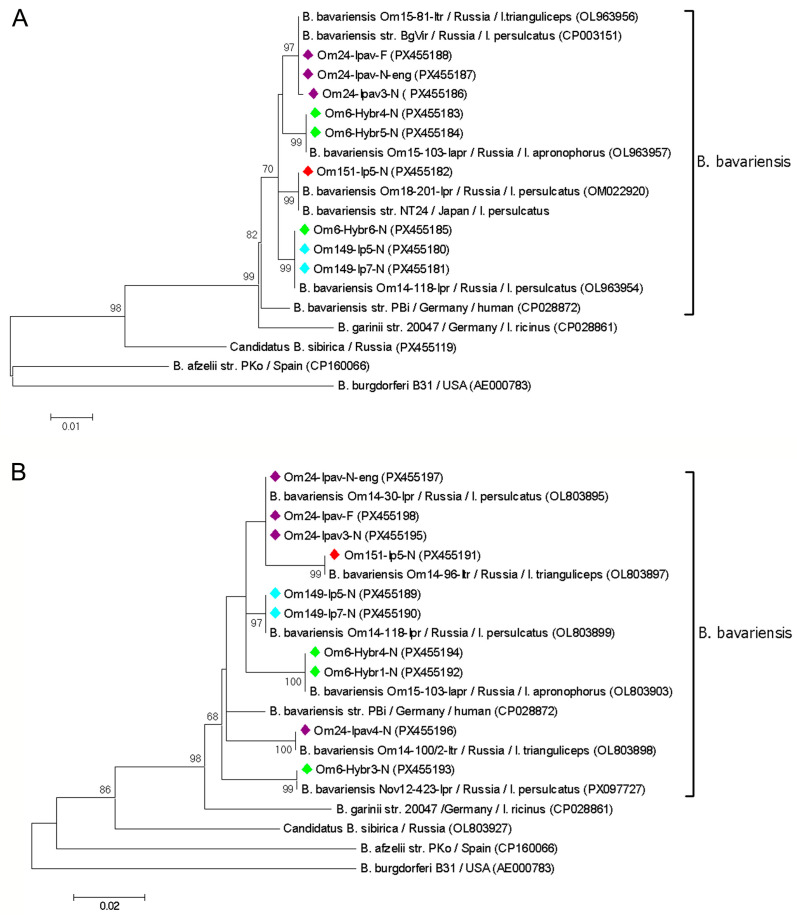
Phylogenetic trees constructed by the ML method based on a 775 bp fragment of the *clp*A gene (**A**) and 366–369 bp fragments of the p83/100 gene (**B**) of *Borrelia bavariensis* identified in molted ticks. Sample names include the vole identifier, tick species, tick identifier, and tick stage. Samples derived from molted ticks that had fed as larvae on the same vole are color-coded. Significant bootstrapping values (>65%) are shown on the nodes. *B. burgdorferi* B31 was used as an outgroup.

All *B. bavariensis* samples detected in individual molted ticks were genotyped based on *clp*A and *p83/100* gene fragments. Among twelve *clp*A gene sequences obtained, three contained polymorphic sites. The remaining nine sequences corresponded to PubMLST alleles 59, 60, 72, and 168, previously identified in *Ixodes* spp. from the Asian part of Russia. Similarly, three of the thirteen *p83/100* gene sequences contained polymorphic sites, while the other ten sequences matched six known variants (OL803899, OL803898, OL803897, OL803895, OL803903, MG010864) from *Ixodes* spp. in Western Siberia. Phylogenetic analysis of both gene fragments demonstrated that in most cases, ticks fed as larvae on the same vole were infected with an identical *B. bavariensis* variant. However, several ticks harbored distinct *B. bavariensis* genovariants that differed from those found in other ticks from the same host (Figure 5).

## 4. Discussion

Lyme borreliosis is the most common tick-borne disease in the Asian part of Russia [43]. Nevertheless, the enzootic cycles of Siberian *B. burgdorferi* s.l. strains, particularly their persistence in vertebrate hosts, remain poorly characterized. To address this knowledge gap, we conducted a comprehensive study of *B. burgdorferi* s.l. in small mammals at a site in the Omsk province of Western Siberia. The sampling area is characterized by high abundances of *I. persulcatus* and *I. trianguliceps* and a low abundance of *I. apronophorus*. Our investigation combined a long-term study of *B. burgdorferi* s.l. prevalence in small mammals captured between 2013 and 2024, analysis of spirochete persistence in naturally infected voles, and evaluation of *Borrelia* transmission from voles to xenodiagnostic ticks.

In all sampling periods, two LB agents, *B. afzelii* and *B. bavariensis*, were detected in small mammals at similar overall prevalence (Table 2). Notably, prevalence varied significantly between sampling periods (from 4.2% to 40.6%), likely due to fluctuations in the abundance and species composition of ticks and small mammals across seasons and years, as previously demonstrated [36]. Similarly, substantial variation in *B. burgdorferi* s.l. prevalence in small mammals (1.9–54.5% in different years) has been observed in the Middle Urals within the *I. persulcatus* distribution area [34]. Notably, the true prevalence of LB agents in voles is likely higher than observed, as our study showed that persisting spirochetes were not detected in all blood samples from infected voles (Figure 2).

In addition to common LB agents, the recently described “*Candidatus* B. sibirica” was detected in a common shrew in 2024. This species had previously been reported in Siberia only in 2015–2017 [33]. Its detection seven years later indicates the presence of a stable enzootic population of “*Candidatus* B. sibirica” in the Omsk province.

This study is the first to investigate the persistence of *B. burgdorferi* s.l. strains from the *I. persulcatus* distribution area in their mammalian hosts. We demonstrated that three genospecies—*B. afzelii*, *B. bavariensis*, and “*Candidatus* B. sibirica”—persisted in voles for the hosts’ entire lifespan, up to 45–50 weeks. The persistence patterns of *B. afzelii* and *B. bavariensis* were similar; both agents were distributed randomly among positive samples, with no observed clearance of either genospecies or replacement of one genospecies by the other (Figure 2). The higher prevalence of *B. bavariensis* in voles captured in September is likely driven by the seasonal dominance of *I. trianguliceps*, a tick species with a suspected association with this genospecies [33,44]. This finding provides the first evidence of long-term persistence for Asian *B. bavariensis* genotypes in small mammals. The observed prolonged persistence of *B. afzelii* is consistent with data from the *I. ricinus* distribution area [17,22,27]. Furthermore, the long-term persistence of “*Candidatus* B. sibirica” in a vole, combined with its initial detection in small mammals and the ticks feeding on them [33], indicates that this candidate species is also maintained in the enzootic cycle with small mammals.

However, prolonged *Borrelia* persistence observed in voles kept in the laboratory may differ from persistence dynamics in natural conditions. It has been suggested that animals under stress can exhibit higher pathogen infection rates due to immunosuppression and reactivation of latent infections [1,45].

In most voles, the simultaneous circulation of several *B. burgdorferi* s.l. isolates, including different genospecies and genovariants within a single species, was observed. These results are not surprising, as the study involved only mature voles that had likely experienced repeated tick exposure prior to capture. In the sampling area, *Ixodes* spp. are known to harbor both *B. afzelii* and *B. bavariensis* [33]. The high genetic diversity of Asian genotypes of *B. bavariensis*, both in this area and in other parts of the *I. persulcatus* distribution range, has been previously reported [6,33,46]. Consistent with findings from ticks, higher genetic variability of *B. bavariensis* compared to *B. afzelii* was found among the isolates persisting in voles, and the identified *B. bavariensis* variants correspond to those previously found in ticks (Figure 3 and Figure 4).

The persistence of *B. burgdorferi* s.l. in individual voles was dynamic, characterized by alternating detection of *B. afzelii* and *B. bavariensis*, either individually or simultaneously. This alternation could result from competition between *Borrelia* species or genovariants, as shown for *B. burgdorferi* s.s. strains [28] or from stochastic variations in spirochete abundance among different genovariants, as demonstrated for multiple clones of a single *B. burgdorferi* s.s. strain persisting in mice [19].

Another possible explanation involves the transformation of spirochetes into dormant forms [47,48], which can revert to active forms, potentially leading to the observed alternation of genovariants. Furthermore, *B. burgdorferi* s.l. can colonize various internal organs in rodents, including the hearts, spleens, joints, and bladder [49,50], from which spirochetes may be periodically released into the bloodstream. This dynamic of persistent infection may also lead to the alternation of *Borrelia* genotypes in peripheral blood. These proposed mechanisms of *Borrelia* persistence could explain our other observation that a substantial subset of PCR-positive voles had only single positive blood samples, whereas in most infected voles the proportion of positive blood samples exceeded 50% (Table 3).

The method of xenodiagnoses, which includes the feeding of laboratory-reared larvae on animals and subsequent analysis of molted ticks for the presence of pathogens, is considered the strongest evidence of reservoir competence of tested animals [1]. To date, in Eurasia, reservoir competence of small mammals for *B. burgdorferi* s.l. has been conclusively demonstrated for rodents within the *I. ricinus* distribution area, but not for those in the *I. persulcatus* range. Specifically, *Apodemus* mice have been confirmed as competent reservoirs for *B. afzelii* and *B. bavariensis* (European genotype), and *Cl. glareolus* for *B. burgdorferi* s.s. and *B. afzelii* [1,10].

In this study, we first showed via xenodiagnosis that all circulating Siberian genospecies of *B. burgdorferi* s.l. can be transmitted to laboratory-reared *I. persulcatus* larvae and survive through at least one molting cycle. Notably, all *Borrelia* species were successfully transmitted after persisting in voles for at least 38 weeks, confirming the long-term persistence of viable spirochetes. The successful transmission of *B. bavariensis*, *B. afzelii*, and “*Candidatus* B. sibirica” was shown for ticks that fed on four, two, and one voles, respectively (Table 4, Figure 2). Unexpectedly, *B. bavariensis* was transmitted by ticks that fed on a vole, with only two blood samples positive for this agent, indicating that even transient spirochetemia can yield viable, infectious pathogens (Figure 2). The obtained results provide the first reliable evidence of reservoir competence of *Cl. rutilus* for *B. bavariensis* (Asian genotypes), *B. afzelii*, and “*Candidatus B. sibirica*”, and of *Cl. rufocanus* for *B. bavariensis* (Asian genotypes).

*Borrelia bavariensis* transmission was demonstrated not only for *I. persulcatus*, but also for *I. pavlovskyi* and interspecies hybrids. Moreover, in one case, *B. bavariensis* was maintained by *I. pavlovskyi* through two consecutive transtadial transmissions—from larva to nymph and from nymph to female. The reliability of these laboratory results was proved by the genetic characterization of all molted nymphs, which confirmed tick species identity and *Borrelia* genospecies. Nevertheless, the obtained data contradict the results of previous field studies from Russian Siberia and the Far East, which reported a low prevalence (1–2%) of *B. bavariensis* in *I. pavlovskyi* adults [29,31,51]. Thus, despite the laboratory-confirmed association between *B. bavariensis* and *I. pavlovskyi*, this association appears to be rare in nature. Similarly, the observed transmission of “*Candidatus* B. sibirica” to *I. persulcatus* is unlikely to be of biological significance, as this candidate species has previously been detected only within the range of *I. apronophorus* [33]. The observed discrepancy between laboratory and field results may be explained by ecological competition, either among *B. burgdorferi* s.l. genospecies or between tick species, which primarily determines pathogen associations in nature.

Notably, the genetic diversity of *B. bavariensis* in molted ticks was lower than in voles (Figure 3, Figure 4 and Figure 5). This may be due to the population “bottlenecks” during spirochete acquisition and tick molting, which could eliminate certain *B. burgdorferi* s.l. genovariants [19,52].

However, the findings regarding the tick-pathogen relationship are preliminary. A limitation of this study is that the effective transmission of *B. bavariensis* to *I. pavlovskyi* and hybrids, and of “*Candidatus* B. sibirica” to *I. persulcatus*, was demonstrated using larvae that fed on a single vole. Furthermore, the analysis of *B. burgdorferi* s.l. transmission in pooled nymphs does not allow an accurate assessment of transmission efficiency. Further experiments using laboratory strains of different *Borrelia* genospecies are required to validate the obtained results and compare transmission efficiency across tick species and bacterial genospecies.

In conclusion, *B. afzelii*, *B. bavariensis*, and, in single cases, “*Candidatus* B. sibirica” can persist in the blood of wild voles during their life. A simultaneous persistence of several *B. burgdorferi* s.l. genospesies or genovariants was found within the same species in most of the infected voles. It was first demonstrated by xenodiagnosis that all genospecies can be effectively transmitted to *I. persulcatus*; in addition, *B. bavariensis* can be transmitted to *I. pavlovskyi* and *I. persulcatus*/*I. pavlovskyi* interspecies hybrids.

## Figures and Tables

**Table 1:** Primers used for identification and genotyping of *Ixodes* spp. and *B. burgdorferi* s.l.

Locus	Organism	Reaction	Primer Name	Primer Sequences 5′-3′	T * (°C)	References
ITS2	Ixodidae	conventional	F-ITS2	cacactgagcacttactctttg	57	[29]
			R1-ITS2	actggatggctccagtattc		
*cox*1	*I. persulcatus*	conventional	Ixodes-F	acctgatatagctttccctcg	55	[29]
			Ipers-R	ttgattcctgttggaacagc		
	*I. pavlovskyi*	conventional	Ixodes-F	acctgatatagctttccctcg	55	[29]
			Ipav-R	taatccccgtggggacg		
IGS	*B. burgdorferi* s.l.	Primary	NC1	cctgttatcattccgaacacag	50	[29]
			NC2	tactccattcggtaatcttggg		
		Nested	NC3	tactgcgagttcgcgggag	50	
			NC4	cctaggcattcaccatagac		
	“*Ca.* B. sibirica”	Nested	Bsib	ataaaacattctaaaaaaatgaaca	50	This study
			NC4	cctaggcattcaccatagac		[29]
clpA	*B. burgdorferi* s.l.	Primary	clpAF1237	aaagatagatttcttccagac	50	[41]
			clpAR2218	gaatttcatctattaaaagctttc		
		Nested	clpAF1255	gacaaagcttttgatattttag	50	
			clpAR2104	caaaaaaaacatcaaattttctatctc		
*p83/100*	*B. burgdorferi* s.l.	Primary	F7	ttcaaagggatactgttagagag	50	[29]
			F10	aagaaggcttatctaatggtgatg		
		Nested	F5	acctggtgatgtaagttctcc	54	
			F12	ctaacctcattgttgttagactt		

* Annealing temperature.

**Table 2:** *Borrelia burgdorferi* s.l. prevalence in the blood of small mammals.

Sampling Periods	No. of Tested Mammals	No (%/95% CI) of Samples Containing DNA of *B. burgdorferi* s.l.	No of Genotyped Samples	No (%/95% CI *) of Samples Containing DNA			
				Ba	Bbav	Bs	Ba + Bbav
June 2013	38	3 (7.9/2.7–20.8)	3	2	1	0	0
September 2013	59	7 (11.9/5.9–22.5)	7	2	5	0	0
July 2014	124	14 (11.3/6.9–18.1)	12	5	5	0	2
September 2014	119	5 (4.2/1.8–9.5)	5	3	1	0	1
July 2015	79	10 (12.7/7.0–21.8)	10	5	2	0	3
September 2015	87	4 (4.6/1.8–11.2)	4	1	2	0	1
September 2016	95	6 (6.3/2.9–13.1)	2	1	1	0	0
June 2017	33	6 (18.2/8.6–34.4)	6	2	4	0	0
October 2017	43	8 (18.6/9.7–32.6)	8	2	6	0	0
September 2018	28	7 (25.0/12.7–43.4)	7	4	3	0	0
August 2024	32	13 (40.6/25.5–57.7)	13	6	5	1	1
**Total**	**737**	**83 (11.3**/**9.2–13.8)**	**77**	**33 (42.9/32.4–54.0)**	**35 (45.5/34.8–56.5)**	**1 (1.3/0.2–7.0)**	**8 (10.4/5.4–19.2)**

%*—of genotyped samples. Abbreviations: Ba—*B. afzelii*, Bbav—*B. bavariensis*, Bs—“*Candidatus* B. sibirica”.

**Table 3:** Persistence of *B. burgdorferi* s.l. in voles captured in July and September 2015.

	Both Sampling Periods	July 2015	September 2015	Statistical Difference Between Sampling Periods
**All examined voles**				
Total number	47	24	23	
No. of males (%/95% CI)	26 (55.3/41.3–68.6)	13 (54.2/35.1–72.1)	13 (56.5/36.8–74.3)	
No. of females (%/95% CI)	21 (44.7/31.4–58.8)	11 (45.8/27.9–64.9)	10 (43.5/25.6–63.2)	
**PCR-positive voles**				
Total number	22	12	10	
No. of males (%/95% CI)	12 (54.6/34.7–73.1)	6 (50.0/25.4–74.6)	6 (60.0/31.3–83.2)	
No. of females (%/95% CI)	10 (45.4/26.9–65.3)	6 (50.0/25.4–74.6)	4 (40.0/16.8–68.7)	
Number (%/95% CI) of voles with a portion of PCR-positive blood samples:				
50–100%	14 (63.6/43.0–80.3)	9 (75/46.8–91.1)	5 (50.0/23.7–76.3)	
15–50%	1 (4.6/0.8–21.8)	0	1 (10.0/1.8–40.4)	
0–15%	7 (31.8/16.3–52.7)	3 (25/8.9–53.2)	4 (40.0/16.8–68.7)	
Number (%/95% CI) of voles with a persistence of:				
*B. bavariensis*	7 (31.8/16.3–52.7)	1 (8.3/1.5–35.4)	6 (60.0/31.3–83.2)	
*B. afzelii*	3 (13.6/4.8–33.3)	2 (16.7/4.7–44.8)	1 (10.0/1.8–40.4)	
*B. bavariensis* + *B. afzelii*	11 (50.0/30.7–69.3)	8 (66.7/39.1–86.2)	3 (30.0/10.8–60.3)	
*B. bavariensis* + *Ca.* B. sibirica	1 (4.6/0.8–21.8)	1 (8.3/1.5–35.4)	0	
**PCR-positive samples**				
Total number	224	142	82	
Number (%/95% CI) of blood samples containing DNA:				
*B. bavariensis*	150 (67.0/60.6–72.8)	80 (56.3/48.1–64.2)	70 (85.4/76.1–91.4)	*χ*^2^ = 19.8, *p* < 0.001
*B. afzelii*	34 (15.2/11.1–20.5)	30 (21.1/15.2–28.6)	4 (4.9/1.9–11.9)	*χ*^2^ = 10.7, *p* = 0.001
*B. bavariensis* + *B. afzelii*	36 (16.1/11.8–21.5)	28 (19.7/14.0–27.0)	8 (9.8/5.0–18.1)	*χ*^2^ = 3.8, *p* = 0.05
*B. bavariensis* + *Ca.* B. sibirica	4 (1.8/0.7–4.5)	4 (2.9/1.1–7.6)	0	

**Table 4:** *Borrelia burgdorferi* s.l. transmission by *Ixodes* spp. larvae.

Voles		Feeding Larvae		Molted Nymphs				MIR */Prevalence # (%/95% CI) of Bbsl, Ba, Bbav, and Bs in Molted Nymphs
ID	Bbsl Species in Voles	Tick Species	Time After Vole Capture (Weeks)	ID	No of Ticks in a Pool	Presence of Bbsl DNA	Bbsl Species in Molted Ticks	
151	Bbav,	Ip	38	151-Ip1	5	+	Bbav, Bs	Bbsl: 21.4/7.6–47.6 *
	Bs	Ip	38	151-Ip3	7	+	Bbav	Bbav: 21.4/7.6–47.6 *
		Ip	38	151-Ip4	1	-	--	Bs: 7.1/1.3–31.5 *
		Ip	38	151-Ip5	1	+	Bbav	
149	Bbav	Ip	38	149-Ip1	5	+	Bbav, Ba	Bbsl: 22.2/10.6–40.8 *
	Ba	Ip	38	149-Ip2	5	+	Bbav, Ba	Bbav: 22.2/10.6–40.8 *
		Ip	38	149-Ip3	7	+	Bbav, Ba	Ba: 11.1/3.9–28.1 *
		Ip	38	149-Ip4	7	+	Bbav	
		Ip	38	149-Ip5	1	+	Bbav	
		Ip	38	149-Ip6	1	-	-	
		Ip	38	149-Ip7	1	+	Bbav	
32-13	Bbav	Ip	38	32-13-Ip1	7	+	Bbav	Bbsl: 18.8/8.9–35.3 *
	Ba	Ip	38	32-13-Ip2	7	+	Bbav	Bbav: 18.8/8.9–35.3 *
		Ip	38	32-13-Ip3	5	+	Bbav	Ba: 6.3/1.7–20.2 *
		Ip	38	32-13-Ip4	5	+	Bbav	
		Ip	42	32-13-Ip5	4	+	Bbav, Ba	
		Ip	42	32-13-Ip6	4	+	Bbav, Ba	
11-13	Bbav	Ip	42	11-13-Ip1	5	-	-	Bbsl: 10.0/1.8–40.4 *
	Ba	Ip	42	11-13-Ip2	5	+	Bbav	Bbav: 10.0/1.8–40.4 *
186	Ba	Ip	35	186-Ip1	5	-	-	NA
		Ip	35	186-Ip2	5	-	-	
		Ip	35	186-Ip3	5	-	-	
187	Bbav	Ip	35	187-Ip1	4	-	-	NA
		Ip	35	187-Ip2	4	-	-	
8-12	-	Ip	42	8-12-Ip1	5	-	-	NA
		Ip	42	8-12-Ip1	5	-	-	
24	Bbav	Ipav	23	24-Ipav1	1	-	-	Bbsl: 50.0/18.8–81.2 #
		Ipav	23	24-Ipav2	1	+	Bbav	Bbav: 50.0/18.8–81.2 #
		Ipav	23	24-Ipav3	1	+	Bbav	
		Ipav	23	24-Ipav4	1	+	Bbav	
		Ipav	23	24-Ipav5	1	-	-	
		Ipav	23	24-Ipav6	1	-	-	
6	Bbav	Hybr	23	6-Hybr1	1	+	Bbav	Bbsl: 71.4/35.9–91.8 #
	Bbav	Hybr	23	6-Hybr2	1	-	-	Bbav: 71.4/35.9–91.8 #
	Bbav	Hybr	23	6-Hybr3	1	+	Bbav	
	Bbav	Hybr	23	6-Hybr4	1	+	Bbav	
	Bbav	Hybr	23	6-Hybr5	1	+	Bbav	
	Bbav	Hybr	23	6-Hybr6	1	+	Bbav	
	Bbav	Hybr	23	6-Hybr7	1	-	-	

Nymphs were analyzed 14 days after molting. MIR *—was calculated for ticks in pools; Prevalence #—was calculated for individual ticks. Abbreviations: Bbsl—*B. burgdorferi* s.l., Ba—*B. afzelii*, Bbav—*B. bavariensis*, Bs—“*Candidatus* B. sibirica”, Ip—*I. persulcatus*, Ipav—*I. pavlovskyi*, Hybr—*I. persulcatus*/*I. pavlovskyi* hybrids.

## Data Availability

The data are contained within the article.

## Supplementary Materials

The following supporting information can be downloaded at: https://www.mdpi.com/article/10.3390/pathogens14121200/s1, Table S1: *Borrelia burgdorferi* s.l. prevalence in the blood of small mammals by seasons.
