# Supplementary material for: The First Study of Borrelia burgdorferi Sensu Lato Persistence in Small Mammals Captured in the Ixodes persulcatus Distribution Area in Western Siberia
Source: Pathogens. 2025 Nov 24;14(12):1200. doi: 10.3390/pathogens14121200 (PMC12735746; doi:10.3390/pathogens14121200)
Supplement: Supplementary file 1 [file pathogens-14-01200-s001.zip › pathogens-3949094-supplementary.pdf]

Table S1. *Borrelia burgdorferi* s.l. prevalence in blood of small mammals by seasons.

Sampling period	Mammalian species	No. of tested mam-mals	No (% / 95% CI) of samples containing DNA of *B. burgdorferi* s.l.	No of typed samp-les	No (%*) of samples containing DNA			
					Ba	Bbav	Bs	Ba+Bbav
**Jun, 2013**	**All species**	**38**	3 (7.9 / 2.7-20.8)	**3**	**2**	**1**	**0**	**0**
	Cl. rutilus	*13*	*1*	*1*	*1*			
	Cl. glareolus	*8*	*1*	*1*	*1*			
	Cl. rufocanus	*17*	*1*	*1*		*1*		
**Sep, 2013**	**All species**	**59**	7 (11.9 / 5.9-22.5)	**7**	**2**	**5**	**0**	**0**
	Cl. rutilus	*15*	*2*	*2*		*2*		
	Cl. glareolus	*26*	*2*	*2*		*2*		
	Cl. rufocanus	*14*	*3*	*3*	*2*	*1*		
	Mi. agrestis	*4*	*0*					
**Jul, 2014**	**All species**	**124**	14 (11.3 / 6.9-18.1)	**12**	**5**	**5**	**0**	**2**
	Cl. rutilus	*61*	*8*	*7*	*2*	*3*		*2*
	Cl. glareolus	*44*	*5*	*4*	*2*	*2*		
	Cl. rufocanus	*19*	*1*	*1*	*1*			
**Sep, 2014**	**All species**	**119**	5 (4.2 / 1.8-9.5)	**5**	**3**	**1**	**0**	**1**
	Cl. rutilus	*62*	*4*	*4*	*2*	*1*		*1*
	Cl. glareolus	*42*	*1*	*1*	*1*			
	Cl. rufocanus	*13*	*0*					
	Mi. agrestis	*2*	*0*					
**Jul, 2015**	**All species**	**79**	10 (12.7 / 7.0-21.8)	**10**	**5**	**2**	**0**	**3**
	Cl. rutilus	*41*	*8*	*8*	*4*	*2*		*2*
	Cl. glareolus	*15*	-					
	Cl. rufocanus	*18*	*2*	*2*	*1*			*1*
	Mi. agrestis	*5*	*0*					
**Sep, 2015**	**All species**	**87**	4 (4.6 / 1.8-11.2)	**4**	**1**	**2**	**0**	**1**
	Cl. rutilus	*46*	*2*	*2*	*1*			*1*
	Cl. glareolus	*25*	*2*	*2*		*2*		
	Cl. rufocanus	*1*	*0*					
	Mi. agrestis	*14*	*0*					
	S. araneus	*1*	*0*					
**Sep, 2016**	**All species**	**95**	6 (6.3 / 2.9-13.1)	**2**	**1**	**1**	**0**	**0**
	Cl. rutilus	*32*	*4*	*2*	*1*	*1*		
	Cl. glareolus	*19*	*2*	*0*				
	Cl. rufocanus	*7*	*0*					
	Mi. agrestis	*37*	*0*					
**Jun, 2017**	**All species**	**33**	6 (18.2 / 8.6-34.4)	**6**	**2**	**4**	**0**	**0**
	Cl. rutilus	*14*	*2*	*2*		*2*		
	Cl. glareolus	*11*	*1*	*1*	*1*			
	Cl. rufocanus	*3*	*2*	*2*		*2*		
	Mi. agrestis	*3*	*0*					
	Mi. oeconomus	*2*	*1*	*1*	*1*			
**Oct, 2017**	**All species**	**43**	8 (18.6 / 9.7-32.6)	**8**	**2**	**6**	**0**	**0**
	Cl. rutilus	*28*	*6*	*6*	*2*	*4*		
	Cl. glareolus	*15*	*2*	*2*		*2*		

**Sep, 2018**	**All species**	**28**	**7 (25.0 / 12.7-43.4)**	**7**	**4**	**3**	**0**	**0**
	Cl. rutilus	15	*3*	*3*	*2*	*1*		
	Cl. glareolus	5	*3*	*3*	*1*	*2*		
	Cl. rufocanus	1	*1*	*1*	*1*			
	Mi. agrestis	3	*0*					
	Ap. agrarius	4	*0*					
**Aug, 2024**	**All species**	**32**	13 (40.6 / 25.5-57.7)	**13**	**6**	**5**	**1**	**1**
	Cl. rutilus	*29*	*11*	*11*	*6*	*4*		*1*
	Mi. agrestis	*2*	*1*	*1*		*1*		
	S. araneus	*1*	*1*	*1*			*1*	
**All periods**	**All species**	**737**	**83 (11.3 / 9.2-13.8)**	**77**	**33 (42.9)**	**35 (45.5)**	**1 (1.3)**	**8 (10.4)**
	Cl. rutilus	*356*	*51 (14.3 / 11.1-18.4)*	*48*	*21 (43.8)*	*20 (41.7)*		*7 (14.6)*
	Cl. glareolus	*210*	*19 (9.0 / 5.9-13.7)*	*16*	*6 (37.5)*	*10 (62.5)*		
	Cl. rufocanus	*93*	*10 (10.8 /6.0-18.7)*	*10*	*5 (50.0)*	*4 (40.0)*		*1 (10.0)*
	Mi. agrestis	*70*	*1 (1.4 / 0.3-7.7)*	*1*		*1*		
	Mi. oeconomus	*2*	*1*	*1*	*1*			
	Ap. agrarius	*4*	*0*					
	S. araneus	*2*	*1*	*1*			*1*	

%*- of genotyped samples. Abbreviations: Ba – *B. afzelii*, Bbav – *B. bavariensis*, Bs – "*Candidatus* B. sibirica".
